# Effects of *TGFBR1* on Proliferation of Dermal Papilla Cells in Fine-Wool Sheep

**DOI:** 10.3390/ani16010036

**Published:** 2025-12-23

**Authors:** Tong Xiao, Yu Luo, Chao Yuan, Yufang Song, Jianxiang Tang, Zengkui Lu, Jianbin Liu, Tingting Guo

**Affiliations:** 1 Lanzhou Institute of Husbandry and Pharmaceutical Sciences of Chinese Academy of Agricultural Sciences, Lanzhou 730050, China; xiaotong3110134994@163.com (T.X.); 17393144478@163.com (Y.L.); yuanchao@caas.cn (C.Y.); songyf805@163.com (Y.S.); 821012450577@caas.cn (J.T.); luzengkui@caas.cn (Z.L.); 2 Sheep Breeding Engineering Technology Research Center of Chinese Academy of Agricultural Sciences, Lanzhou 730050, China; 3 Key Laboratory of Animal Genetics and Breeding on Tibetan Plateau, Ministry of Agriculture and Rural Affairs, Lanzhou 730050, China

**Keywords:** fine-wool sheep, dermal papilla cells, *TGFBR1*, hair follicle, single-cell transcriptomics

## Abstract

Dermal papilla cells (DPCs) serve as the signaling hub regulating hair follicle (HF) development and cyclical growth. This study aims to investigate the biological function and molecular mechanisms of *TGFBR1* (transforming growth factor β receptor 1), a differentially expressed gene identified through single-cell transcriptomic sequencing (scRNA-seq) in the DPCs from fine-wool sheep. Primary DPCs were isolated and purified using a combination of enzymatic digestion and mechanical dissociation, followed by immunofluorescence identification (α-SMA and SOX2-positive). Following successful transfection with constructed *TGFBR1* overexpression plasmids and siRNA interference vectors, cell proliferation was assessed via EDU staining and CCK-8 assays. mRNA expression of key genes in Wnt/β-catenin, BMP, and Notch signaling pathways (*PCNA*, *CCND1*, *CTNNB1*, *SFRP2*, *BMP2*, *NOTCH3*, *SMAD4*, etc.) was validated by RT-qPCR. Single-cell transcriptomics revealed significant downregulation of *TGFBR1* in DPCs from fine-wool sheep. Functional validation demonstrated that *TGFBR1* overexpression markedly suppressed DPC proliferation, whereas knockdown of *TGFBR1* expression promoted DPC proliferation. Molecular mechanism studies showed that *TGFBR1* overexpression significantly downregulated *PCNA*, *CCND1*, *CTNNB1*, *NOTCH3*, and *SMAD4* while upregulating *SFRP2*, *BMP2*, and *TGFB1* expression. These findings demonstrate that *TGFBR1* acts as a negative regulator of DPCs proliferation by modulating the activity of multiple signaling pathways, including Wnt/β-catenin, BMP, and Notch, thereby suppressing the proliferative capacity of DPCs. This study not only provides new theoretical support for elucidating the role of the TGF-β signaling pathway in H development but also offers theoretical reference for in-depth research on molecular breeding in ultra -fine-wool sheep and the molecular mechanisms underlying HF development.

## Simple Summary

This study investigates the regulation of dermal papilla cells proliferation by *TGFBR1*, a differentially expressed gene identified through single-cell transcriptomic sequencing. The aim is to elucidate the molecular mechanisms through which *TGFBR1* governs dermal papilla cells and the role of *TGFBR1* in hair follicle development of fine-wool sheep. Single-cell transcriptomic sequencing data revealed significant downregulation of *TGFBR1* in dermal papilla cells of ultra-fine-wool sheep, suggesting that this gene serves as a key negative regulator for wool fiber diameter. To validate this hypothesis, we constructed *TGFBR1* overexpression plasmids and knockdown vectors for transfection into dermal papilla cells. Combined with functional assays such as EDU and CCK-8, we confirmed *TGFBR1* exerts negative control over dermal papilla cell proliferation. Mechanistic studies revealed that *TGFBR1* modulates the activity of multiple signaling pathways, including Wnt/β-catenin, BMP, and Notch, thereby influencing the proliferative capacity of fine-wool dermal papilla cells and hair follicle development. These findings provide valuable targets and theoretical references for developing molecular breeding strategies for fine-wool sheep.

## 1. Introduction

Wool, as a vital natural textile material, possesses excellent biodegradability and renewability. Its unique biological properties, such as thermal insulation and felting capability, make it highly valued in the textile industry, conferring significant economic value [1]. As DPCs are the biological “production factories” of wool fibers, the structure and functional state of DPCs directly determine key physical properties such as fiber diameter (FD) and curvature. Among the diverse cellular components of the HFs, DPCs specialized mesenchymal cells located at the basal region of HFs play a central role in follicular morphogenesis, cyclic growth, and developmental regulation. They serve as the “signaling hub”, initiating and sustaining the hair cycle, releasing multiple growth factors via paracrine mechanisms, while activating key signaling pathways to precisely regulate follicular development and structural homeostasis [2]. Research indicates that the number and volume of DPCs are closely correlated with the FD, curvature morphology, and cyclical growth characteristics of HFs. When the number of DPCs falls below a critical threshold, even intact epithelial structures cannot initiate new hair growth, leading to follicular dysfunction [3]. Therefore, in-depth analysis of the biological characteristics of DPCs and their molecular regulatory mechanisms is crucial for understanding HF development and wool trait formation.

*TGFBR1* is a key member of the serine/threonine protein kinase family, playing a vital role in the TGF-β/Smad signaling pathway. Its primary function is to mediate intracellular signal transduction as a transmembrane receptor [4]. By recognizing and binding the TGF-β ligand, this receptor translates extracellular signals into intracellular cascades, thereby extensively regulating key biological processes such as cell proliferation, migration, differentiation, and apoptosis [5,6]. Beyond activating the classical Smad-dependent pathway, *TGFBR1* can activate other signaling pathways through non-canonical mechanisms, such as MAPK (mitogen-activated protein kinase) [7], PI3K (phosphatidylinositol-3-kinase) [8], and AKT (A serine/threonine kinase) [9], demonstrating its multifunctionality and complexity within cellular signaling networks. Numerous studies have elucidated *TGFBR1*’s regulatory functions across different cell types. For instance, all-trans retinoic acid (ATRA) reduces DPC proliferation and induces apoptosis via the TGF-β/Smad pathway, thereby influencing HF development [10]. MIR-22 regulates the downstream of smad3 signaling pathway by targeting *TGFBR1*, thereby inhibiting myoblast proliferation and promoting differentiation [11]. Knockdown of *TGFBR1* in ST2 cells accelerates adipogenic differentiation [12]; Let-7a inhibits cell proliferation by targeting *TGFBR1* in cervical carcinoma [13]. These studies collectively demonstrate that *TGFBR1*, as a key regulatory node, broadly participates in the molecular regulation of diverse cellular fate decisions. However, the specific biological mechanisms of this gene in DPC proliferation and differentiation remain unclear and warrant further systematic investigation.

Based on the aforementioned research background, this study uses fine-wool sheep as experimental subjects to systematically elucidate the biological functions of *TGFBR1* in HF development. First, tissue localization analysis clarifies the expression characteristics of *TGFBR1* for HF structures in skin. Subsequently, molecular biological techniques such as gene knockdown and overexpression are employed to regulate *TGFBR1* expression levels in in vitro cultured DPCs, investigating its effects on key biological processes including DPCs proliferation and activity. Furthermore, by integrating studies on HF development-related signaling pathways, we elucidate the molecular mechanisms through which *TGFBR1* participates in regulating DPC proliferation and HF morphogenesis. This research not only provides new experimental evidence for understanding the role of the TGF-β family in HF development but also lays a theoretical foundation for investigating the molecular regulatory networks governing HF development in fine-wool sheep breeding.

## 2. Materials and Methods

### 2.1. Animal Tissue Samples

All experimental animals used in this study were sourced from the Fine-Wool Sheep Core Breeding Farm (Gansu Provincial Sheep Breeding Technology Promotion Station, Sunan, Gansu, China). Under identical rearing conditions, six 14-month-old Alpine Merino ewes of the same paternal lineage were selected and divided into three groups of two each based on wool count and FD, an ultra-fine group (FD: <18 μm), a medium-fine group (FD: 18.1~20.0 μm), and a fine group (FD: 20.1~21.5 μm), for sampling. Skin tissue samples (0.5 cm × 0.5 cm) were collected from the scapular region of the experimental sheep using a skin biopsy punch (ACUDERM, Fort Lauderdale, FL, USA). Blood was rinsed off the tissue with PBS, and samples were preserved in PBS containing penicillin and streptomycin. Cell isolation experiments were conducted immediately upon arrival at the laboratory. The animal study protocol was approved by the Institutional Ethics Committee of the Institute of Animal Sciences, Chinese Academy of Agricultural Sciences (IAS-CAAS) (permit no. SYXK-2014-0002). Approval date: 13 May 2022.

### 2.2. ScRNA-Seq Data Analysis

ScRNA-seq data were generated using the 10× Genomics platform with Illumina Nova PE150 sequencing (Illumina, San Diego, CA, USA). Raw data underwent quality control using Cell Ranger (v.3.0), followed by clustering analysis with Seurat (v.5.2.0) and differential gene expression analysis. This study focused on cell lineages harboring LUM-labeled [14] and APOD-labeled [15] DPCs. *TGFBR1*, the gene selected for validation, was identified as a downregulated gene in the intergroup difference between ultra-fine group and fine group.

### 2.3. Isolation, Purification, and Immunofluorescence of DPCs

Under aseptic conditions, we collected a 0.5 cm × 0.5 cm skin tissue sample from the posterior margin of the shoulder blade of Alpine Merino sheep. DPCs were isolated using enzymatic digestion and mechanical dissociation. The skin tissue sample was immersed in 75% ethanol for 30 s, then rinsed with 10× PBS and 1× PBS separately for 3~5 min each. Wool and adipose tissue were trimmed from the skin using sterile surgical scissors. The tissue was then dissected into small pieces along the direction of HF growth and digested in 0.25% trypsin solution at 37 °C for 1.5~2 h. Once softened, we used a 1 mL syringe to isolate individual HF units. We incised the terminal bulbus pilosus and extruded the dermal papilla. We transferred the dermal papilla to a 48-well plate for culture using a pipette tip. Within 6~7 days, DPCs emerged from the papilla and adhered to the culture surface. Once densely populated, the cells were digested and passaged. Leveraging their adherent nature, DPCs were purified using differential digestion. After three consecutive purifications, DPCs more than 95% pure were obtained.

Identification of DPC marker proteins α-SMA and SOX2 was performed using immunofluorescence staining. Fourth or fifth -generation DPCs were seeded onto plates. When cells reached 60~70% confluence, medium was removed, cells were washed three times with PBS, and cells were fixed with 4% paraformaldehyde for 20 min. After three PBS washes, cells were permeabilized with 0.5% Triton X-100 for 10 min, followed by three PBS washes and blocking with 3% BSA for 1 h. After three PBS washes, we added 1:100 diluted primary antibody specific to the marker protein and incubated the cells overnight at 4 °C. We washed the cells three times with PBS, then added 1:100 diluted Cy3-labeled secondary antibody (goat anti-rabbit) (Biyun Tian, Shanghai, China) and incubated the cells for 1 h at room temperature in the dark. Finally, we washed the plates three times with PBS, stained the cell nuclei with DAPI at room temperature for 10 min, and obtained images using a confocal microscope. All cell cultures were performed at 37 °C in a 5% CO_2_ incubator.

### 2.4. Construction of Overexpression and Interference Vectors

Based on the *TGFBR1* gene coding sequence published in the NCBI database, gene-specific primers containing double restriction sites for HindIII and EcoRI were designed. The products were directionally cloned into the eukaryotic expression vector to construct the pcDNA3.1(+) recombinant plasmid. The negative control vector pcDNA3.1-NC, recombinant plasmid pcDNA3.1-*TGFBR1*, and siRNA-*TGFBR1* were designed and synthesized by GenePharma (Suzhou, China). Sequence information is shown in Table 1.

### 2.5. Real-Time Fluorescent Quantitative PCR (RT-qPCR)

Total RNA was extracted from DPCs using the Trizol method, with total RNA concentration measured by UV spectrophotometry (IMPLEN, Palo Alto, CA, USA). Total RNA samples extracted from DPCs following successful transfection (Zeta Life, San Francisco, CA, USA) were reverse-transcribed using a Prime Script™ RT Reagent Kit with gDNA Eraser (Vazyme, Nanjing, China) to obtain cDNA fragments. RT-qPCR primers were designed using NCBI Primer-Blast based on gene coding sequences published in the NCBI database. Primers were synthesized by Sangon Biotech Co., Ltd. (Shanghai, China) (Table 2). Reaction reagents were added to RNAse-free Eppendorf tubes, with β-actin serving as the internal control. Reaction protocol was 94 °C pre-denaturation for 30 s, 94 °C denaturation for 10 s, and 60 °C extension for 30 s, repeated for 40 cycles.

### 2.6. Cell Proliferation and Viability Assays

EDU proliferation assay: DPCs were cultured until they reach a well-established, morphologically stable state. Cell proliferation was assessed using an EDU assay kit (Biyun Tian, China) 24 h after transfection with overexpression plasmids and siRNA. The EDU solution was diluted in medium to a 1:500 ratio. We added equal volumes to 6-well plates in a 1:1 ratio, incubated the plates for 2 h, aspirated the medium, and added 4% paraformaldehyde for 15 min at room temperature. After discarding the fixative, we washed the plates 3 times with 3% BSA-PBS solution for 3~5 min. We added 0.3% Triton X-100 permeabilization solution and incubated the plates for 15 min. The plates were thoroughly washed, then 500 μL Click reaction solution was added to each well. We gently shook the plate to ensure complete mixing of medium and reaction solution. We incubated the plate at room temperature in the dark for 30 min. After incubation, the plate was washed 3 times. We added Hoechst 33,342 reaction solution to the cells for nuclear staining. After 10 min, we observed cell proliferation under a fluorescence microscope and document images.

CCK-8 assay: Transfected DPCs were seeded into 96-well plates with three biological replicates per group. CCK-8 solution was added in the dark. After incubation at 37 °C in a 5% CO_2_ incubator for 2, 12, 24, 36, and 48 h, absorbance was measured at 450 nm.

### 2.7. Data Statistics and Analysis

RT-PCR results were analyzed for relative quantitative analysis by 2^−ΔΔCt^ method. One-way ANOVA conducted with SPSS 22.0 (IBM, Armonk, NY, USA) software was used for statistical analysis. Experiments were designed with at least three biological replicates. All results are expressed as mean ± standard deviation (mean ± SD). * *p* < 0.05, ** *p* < 0.01, and *** *p* < 0.001 indicate statistical significance. All bar charts were generated using GraphPad Prism 10.0.

## 3. Results

### 3.1. Differentially Expressed Genes from DPCs in Ultra-Fine Group and Fine Group of Fine-Wool Sheep

**Figure 1:**
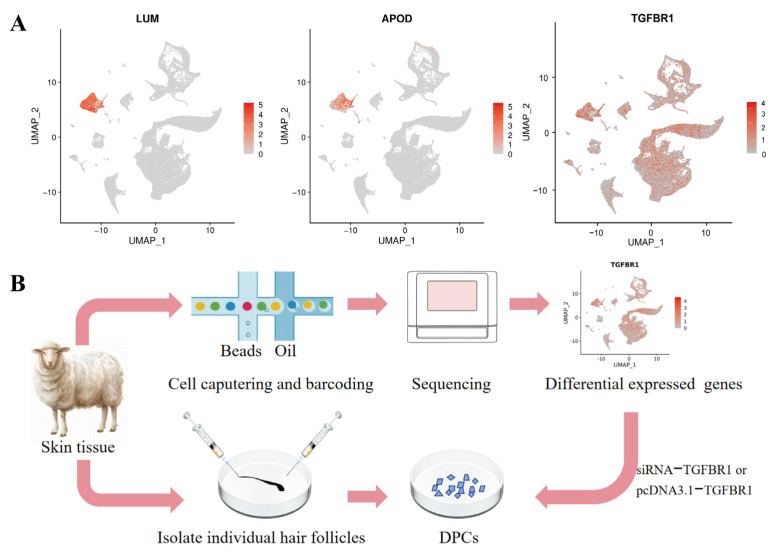
Screening and localization of differentially expressed genes in DPCs in ultra-fine group and fine group of fine-wool sheep. (**A**) Expression localization of *LUM*, *APOD*, and *TGFBR1* in scRNA-seq analysis. Note: *LUM* is a marker gene for dermal cells; *APOD* is a marker gene for DPCs; (**B**) experimental workflow diagram of this study.

ScRNA-seq analysis revealed 147 significantly differentially expressed genes between the two DPC populations, comprising 71 significantly upregulated genes and 76 significantly downregulated genes (Table S1). UMAP visualization revealed that cell regions highly expressing *LUM* and *APOD* could be identified as DPCs (Figure 1A). Notably, among the differentially expressed genes, *TGFBR1* exhibited widespread expression in the hair papilla region, suggesting its role in regulating DPC function.

### 3.2. Isolation, Purification, and Identification of DPCs

**Figure 2:**
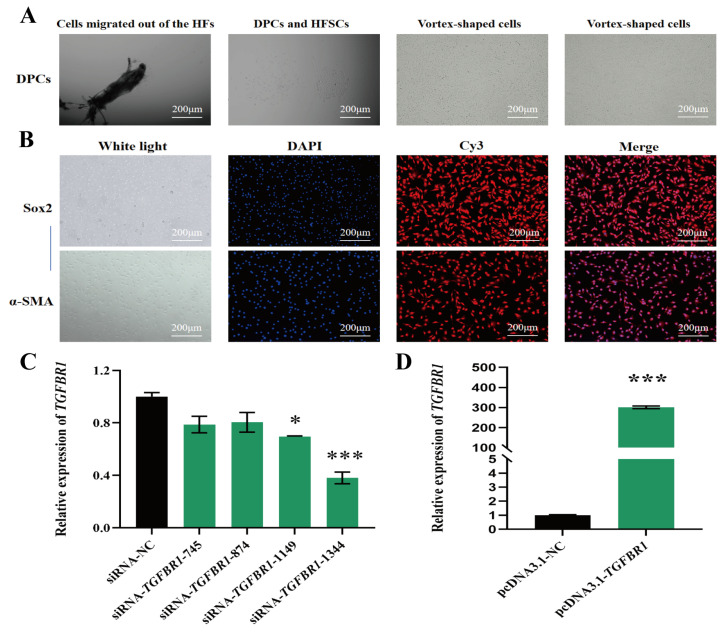
Purification and identification of DPCs and the effect of *TGFBR1* on their proliferation. (**A**) DPCs and HFSCs isolated from HFs, showing migrating and vortex-cultured DPCs; (**B**) immunofluorescence showing expression of α-SMA and SOX2 in isolated DPCs, where blue fluorescence indicates nuclear staining, and red fluorescence indicates target proteins; (**C**) expression of *TGFBR1* interference fragment in DPCs after transfection; (**D**) expression of *TGFBR1* overexpression vector in DPCs after transfection. * *p* < 0.05, and *** *p* < 0.001.

This study successfully isolated primary DPCs from fine-wool sheep using a combination of mechanical separation and enzymatic digestion, establishing a stable in vitro culture system (Figure 1B). Between 6 and 7 days post-follicle isolation, DPCs were observed migrating from the dermal papilla and adhering to the bottom of the culture dish. Their initial morphology was predominantly triangular, with plump cell bodies and abundant cytoplasm. As culture duration increased, cell morphology gradually changed, losing the initial triangular shape. The first passage was performed after around 10 days of culture. In the primary cell culture, in addition to the target DPCs, there were mixed populations of hair follicle stem cells (HFSCs) with a pebble-like morphology and long spindle-shaped fibroblast-like cells. After morphological stabilization, differential digestion ultimately yielded DPCs exhibiting stable growth characteristics, irregular spindle-shaped morphology, and characteristic vortex-like arrangement (Figure 2A). Immunofluorescence results revealed the significant positive expression of both markers in the cultured cells, thereby further confirming that the isolated cells were DPCs that were suitable for subsequent functional validation experiments (Figure 2B).

### 3.3. TGFBR1 Inhibits Proliferation of DPCs

RT-qPCR results showed that compared with the control and the empty vector control groups, *TGFBR1* mRNA expression levels were significantly elevated in the overexpression group, confirming successful establishment of the overexpression system (Figure 2D). Among the four screened siRNA interference fragments, each siRNA exhibited varying degrees of reduced expression. However, *TGFBR1*-siRNA-1344 demonstrated the most pronounced knockdown efficiency, significantly downregulating *TGFBR1* transcription levels. Consequently, *TGFBR1*-siRNA-1344 was selected as the effective interference tool for subsequent functional experiments (Figure 2C).

**Figure 3:**
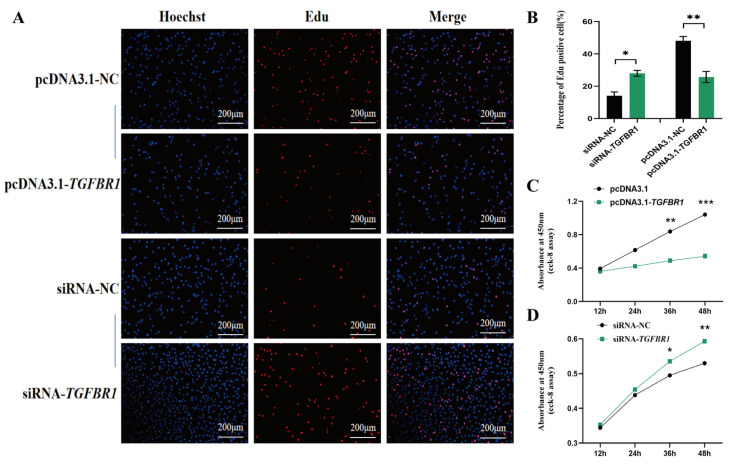
Effects of *TGFBR1* on DPC proliferation. (**A**) Effects of *TGFBR1* overexpression and knockdown on DPC proliferation. Red fluorescence indicates EDU; blue fluorescence indicates Hoechst staining. (**B**) EDU-positive cell rates after *TGFBR1* overexpression and knockdown. (**C**) OD value curves for *TGFBR1* overexpression at 12 h, 24 h, 36 h, and 48 h; (**D**) OD value curves for *TGFBR1* knockdown at 12 h, 24 h, 36 h, and 48 h.* *p* < 0.05, ** *p* < 0.01, and *** *p* < 0.001.

EDU assay results revealed that *TGFBR1* overexpression significantly reduced the EDU-positive cell ratio, indicating marked inhibition of DPCs proliferation. Consistent with this, CCK-8 assays showed lower absorbance values in the overexpression group at all time points compared to the control group, further confirming *TGFBR1*’s inhibitory effect on DPC proliferation (Figure 3A–C). Conversely, after *TGFBR1* knockdown, the EDU-positive cell ratio significantly increased, and CCK-8 assay results similarly demonstrated enhanced cell proliferation activity (Figure 3A,B,D).

### 3.4. TGFBR1 Regulates Genes Associated with HF Development

**Figure 4:**
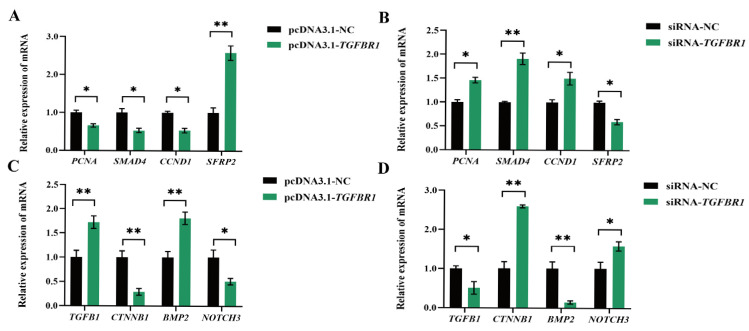
Effects of *TGFBR1* on genes related to HF development. (**A**) Effects of *TGFBR1* overexpression on *PCNA*, *SMAD4*, *CCND1*, and *SFRP2* expression; (**B**) effects of *TGFBR1* knockdown on *PCNA*, *SMAD4*, *CCND1*, and *SFRP2* expression; (**C**) effects of *TGFBR1* overexpression on *TGFB1*, *CTNNB1*, *BMP2*, and *NOTCH3* expression; (**D**) effects of *TGFBR1* knockdown on *TGFB1*, *CTNNB1*, *BMP2*, and *NOTCH3* expressions. * *p* < 0.05, ** *p* < 0.01.

Experimental results demonstrated that *TGFBR1* overexpression in DPCs significantly downregulated *PCNA* and *CCND1* transcription levels. Concurrently, mRNA expressions of *CTNNB1*, *NOTCH3*, and *SMAD4* were markedly suppressed. Correspondingly, the gene expressions of *SFRP2*, *BMP2*, and *TGFB1* were significantly upregulated (Figure 4A,C). This expression pattern was perfectly validated in the reverse experiment: upon *TGFBR1* knockdown, the suppressed genes (*PCNA*, *CCND1*, *CTNNB1*, *NOTCH3*, *SMAD4*) showed significantly increased expression, while the activated genes (*SFRP2*, *BMP2*, *TGFB1*) exhibited markedly decreased expression (Figure 4B,D).

## 4. Discussion

As vital skin appendages, HFs serve as the fundamental biological basis for regulating hair formation and its key economic traits (e.g., diameter, curvature). Their morphogenesis and cyclical growth depend on intricate collaboration among multiple cell types, including DPCs, HFSCs, and matrix cells (Mxs) [16]. During embryonic development, HF formation relies on a series of interactions between the epidermal basal layer and dermal mesenchyme. Postnatally, localized mesenchymal cell clusters gradually differentiate into DPCs, which are subsequently enveloped by Mxs. These cells persist as DPCs, continuously influencing HF growth and development [17]. Furthermore, this process is tightly regulated by multiple signaling pathways, including the Wnt/β-catenin pathway, which promotes HF initiation and growth [18,19]; the BMP signaling pathway, involved in cell fate determination [20,21]; the SHH signaling pathway, which regulates morphogenesis [22]; the Notch signaling pathway, affecting stem cell maintenance and differentiation [23,24]; and the transforming growth factor TGF-β family, which is extensively involved in regulating cellular behavior [25]. These pathways form a complex regulatory network through paracrine and autocrine mechanisms, collectively determining the developmental progression and homeostasis of HFs. *TGFBR1*, as the specific receptor for the TGF-β signaling pathway, plays crucial roles in diverse tissues and cells. Typically, TGF-β ligand binding to its receptor activates downstream Smad proteins, thereby regulating target gene expression and influencing cellular processes including proliferation, differentiation, and apoptosis [26]. Studies indicate that *TGFBR1* mediates Smad phosphorylation and downstream signaling to influence myoblast proliferation and differentiation [27]. Furthermore, microRNA-98 suppresses cardiac fibroblast differentiation by targeting *TGFBR1*, highlighting the receptor’s broad role in cell fate determination [28]. However, although the functions of *TGFBR1* in various biological systems are increasingly well-defined, its specific mechanism of action in DPCs in fine-wool sheep remains to be systematically elucidated.

This study functionally validated *TGFBR1* at the cellular level, which is a differentially downregulated gene in DPCs that was identified through scRNA-seq. The proliferative capacity of DPCs is fundamental to maintaining their population size and performing their functions, being crucial for the normal growth and development of HFs as well as their cyclical growth [29]. EDU and CCK-8 assays demonstrated that *TGFBR1* expression directly influences DPCs proliferation capacity: overexpression significantly suppressed DPC proliferation, manifested as reduced EDU-positive cell proportions and decreased CCK-8 absorbance values. Conversely, knocking down *TGFBR1* expression markedly enhanced cellular proliferation, suggesting *TGFBR1* acts as a negative regulator of DPC proliferation. This further supports the critical role of the TGF-β signaling pathway in initiating the HF regression phase or maintaining the quiescent state of DPCs during the resting phase. Furthermore, the skin samples used for scRNA-seq in this study were collected in August, when the HFs of fine-wool sheep were in a vigorous growth phase. The differential downregulation of *TGFBR1* in DPCs from ultra-fine-wool sheep aligns with its proliferation-inhibiting effect observed in functional validation experiments, providing additional evidence for the gene’s negative regulatory role in DPC growth from a natural expression pattern perspective.

RT-qPCR results for HF-development-related genes revealed a potential mechanism through which *TGFBR1* influences DPC proliferation. *PCNA*, a classic marker assessing cell proliferation capacity [30], exhibited significantly reduced mRNA expression following *TGFBR1* overexpression, providing molecular evidence for *TGFBR1*’s inhibitory effect on DPCs proliferation. The Wnt/β-catenin signaling pathway plays a crucial role in DPC proliferation and differentiation [31]. *CNND1* and *CTNNB1*, the core downstream effectors promoting HF formation and development [32], both exhibited significantly downregulated mRNA expression following *TGFBR1* overexpression, suggesting *TGFBR1* influences Wnt/β-catenin signaling by suppressing downstream gene expression, thereby affecting DPC proliferation. *SFRP2*, a regulator of the Wnt pathway, plays a role in suppressing HF growth, degeneration, and remodeling by inhibiting keratinocyte proliferation [33]. Its upregulation following *TGFBR1* overexpression provides further compelling evidence that *TGFBR1* influences the Wnt/β-catenin signaling pathway. *TGFB1*, belonging to the same transforming growth factor β family as *TGFBR1*, inhibits HF epithelial cell growth and induces cell degeneration [34]. Its expression increases upon *TGFBR1* overexpression, consistent with *TGFB1*’s known role in suppressing HF epithelial cell proliferation, further reinforcing *TGFBR1*’s proliferative inhibitory function. The BMP signaling pathway regulates cell proliferation, differentiation, and apoptosis in multiple organs, including skin [35]. In cashmere goat HFs, *BMP2* is highly expressed during the resting phase but is barely detected during the active phase [36]. This finding further supports the hypothesis that *TGFBR1* promotes the transition of DPCs to a quiescent state. *SMAD4* is a key regulator of HF formation and development, synergistically modulating epidermal homeostasis via the TGF-β pathway [37], maintaining the morphological and structural integrity of HFSCs [38], and promoting DPC proliferation [39]. The Notch signaling pathway suppresses cell differentiation to promote proliferation in HFs, thereby maintaining stem cell characteristics, as a key component of this pathway. The *NOTCH3* gene likely plays a crucial role in this process [40]. The significant reduction in *SMAD4* and *NOTCH3* following *TGFBR1* overexpression further supports *TGFBR1*’s negative regulatory role in DPCs. Collectively, these findings indicate that *TGFBR1* coordinates multiple signaling pathways, including Wnt/β-catenin, BMP, and Notch, to form a complex molecular network that finely regulates DPC proliferation capacity.

## 5. Conclusions

This study demonstrates that *TGFBR1* is expressed in DPCs and inhibits DPC proliferation. Further mechanistic analysis revealed that *TGFBR1* exerts this effect by modulating key signaling pathways, including Wnt/β-catenin, BMP, and Notch. These results identify *TGFBR1* as a key negative regulator of DPC proliferation and provide new insights into the molecular mechanisms of hair follicle development. The findings offer potential implications for the genetic improvement of wool traits.

## Figures and Tables

**Table 1:** siRNA sequence information.

Gene Name	Sequence (5′-3′)
siRNA-*TGFBR1*-1344	GUUGCCUUAUUAUGAUCUUTT
	AAGAUCAUAAUAAGGCAACTT
siRNA-*TGFBR1*-1149	GGCCACAGAUACAAUUGACTT
	GUCAAUUGUAUCUGUGGCCTT
siRNA-*TGFBR1*-745	GGAGAAGAAGUUGCUGUUATT
	UAACAGCAACUUCUUCUCCTT
siRNA-*TGFBR1*-874	GACAAUGGCACAUGGACUCTT
	GAGUCCAUGUGCCAUUGUCTT
siRNA-NC	UUCUCCGAACGUGUCACGUTT
	ACGUGACACGUUCGGAGAATT

**Table 2:** RT-qPCR primers.

Gene Name	Forward Primer Sequence	Reverse Primer Sequence
*SFRP2*	GACAACGACCTTTGCATCCC	ATACCTTCGGAGCTTCCTCG
*CTNNB1*	AAGACATCACTGAGCCTGCC	GTCCGTAGTGAAGGCGAACA
*BMP2*	CACACCCTACCCGAGATTGG	CTGAGTCCCCAGTAATCCGC
*PCNA*	CGTGAACCTCACCAGCATGTC	GTGTCCGCATTATCTTCAGCC
*β-actin*	CAGTCGGTTGGATGGAGCAT	AGGCAGGGACTTCCTGTAAC
*TGFBR1*	TCCAACTGTCGGAAAGCCG	TGGTGAATGACAGTGCGGTT
*CCND1*	GCACGACTTCATCGAGCACT	ATGAACTTCACGTCTGTGGC
*SAMD4*	GTCAGTGTCACCGCCAGATG	AGCAGCTGACAAACTGATGGC
*NOTCH3*	CTTGGGTCCTGTGGTGAGTC	AGCAGGAGGAGTGAGAGAGG
*TGFB1*	ACACACAGTACAGCAAGGTCC	CACGTAGTACACGATGGGCA

## Data Availability

All data are presented in this article.

## Supplementary Materials

The following supporting information can be downloaded at: https://www.mdpi.com/article/10.3390/ani16010036/s1, Table S1: Differentially expressed genes between the ultra-fine and fine groups.

## Abbreviations

The following abbreviations are used in this manuscript:

HFs	Hair follicles
DPCs	Dermal papilla cells
HFSCs	Hair follicle stem cells
TGFBR1	Transforming growth factor β receptor 1
ScRNA-seq	Single-cell transcriptomic sequencing
FD	Fiber diameter
MAPK	Mitogen-activated protein kinase
PI3K	Phosphatidylinositol-3-kinase
AKT	A serine/threonine kinase
ATRA	All-trans retinoic acid
Mxs	Matrix cells
